# Necrostatin-1 Alleviates Lung Ischemia-Reperfusion Injury via Inhibiting Necroptosis and Apoptosis of Lung Epithelial Cells

**DOI:** 10.3390/cells11193139

**Published:** 2022-10-06

**Authors:** Lingjun Dong, Fuxiang Liang, Zhiling Lou, Yangfan Li, Jinsheng Li, Yaling Chen, Jingjing Ding, Bin Jiang, Chuanqiang Wu, Huan Yu, Yafei Liu, Weiping Zhang, Yunbi Lu, Ming Wu

**Affiliations:** 1 Department of Thoracic Surgery, The Second Affiliated Hospital, Zhejiang University School of Medicine, Hangzhou 310009, China; 2 Department of Pharmacology, School of Basic Medical Sciences, Zhejiang University, Hangzhou 310058, China; 3 Key Laboratory of The Diagnosis and Treatment of Severe Trauma and Burn of Zhejiang Province, Hangzhou 310009, China

**Keywords:** necrostatin-1, necroptosis, apoptosis, lung ischemia-reperfusion injury, lung epithelial cells

## Abstract

Lung ischemia-reperfusion injury (LIRI) is associated with many diseases, including primary graft dysfunction after lung transplantation, and has no specific and effective therapies. Necroptosis contributes to the pathogenesis of ischemia-reperfusion injury. Necrostatin-1 (Nec-1), the necroptosis inhibitor targeting RIPK1, has been reported to alleviate ischemia-reperfusion injury in various organs. However, the underlying mechanism of Nec-1 in LIRI remains unclear. In this paper, an in vivo LIRI model was built up by left lung hilar clamping in mice, and an in vitro cold ischemia-reperfusion (CI/R) model using BEAS-2B cells was applied to mimic the lung transplantation setting. We found Nec-1 significantly alleviated ischemia-reperfusion-induced lung injury, cytokine releasing, and necroptosis of epithelial cells in mouse lungs. In vitro, Nec-1 also mitigated CI/R-induced cell death and inflammatory responses in BEAS-2B cells, and these protective effects were achieved by simultaneously inhibiting the formation of necrosome and RIPK1-dependent apoptosis. However, Nec-1 decreased the necrosome number but increased the apoptosis level in lung tissues after ischemia reperfusion. We further clarified that Nec-1 could also attenuate lung injury by promoting neutrophil apoptosis from flow cytometry. In conclusion, Nec-1 alleviated lung ischemia-reperfusion injury by inhibiting necroptosis and apoptosis of epithelial cells and promoting the apoptosis of neutrophils. Thus, Nec-1 could be a promising medication against primary graft dysfunction after lung transplantation.

## 1. Introduction

Lung transplantation remains the only definitive treatment for end-stage pulmonary diseases. Primary graft failure (PGD), characterized by post-transplant hypoxemia, pulmonary edema, and bilateral lung infiltrates on radiography, is the most common cause of early death and chronic lung graft dysfunction [1]. Despite significant advances in organ protection and perioperative support for lung transplant recipients, up to 30% of patients still develop PGD after lung transplantation [2]. Lung ischemia-reperfusion (IR) injury (LIRI) is recognized as the primary cause of PGD [3]. LIRI is also associated with many other clinical situations, including cardiopulmonary bypass, single-lung ventilation, and pulmonary embolism. To date, the exact mechanism of LIRI is still not fully understood, and no specific agents are used clinically to treat LIRI [4].

Necrotic cell death plays a dominant role in LIRI and is closely related to the long-term prognosis of lung transplantation [5]. However, the potential for the intervention of necrotic cell death was overlooked for a long time because necrosis was considered unregulated. Recent studies changed this idea and partially revealed the specific signal pathway of different types of regulated necrosis, including necroptosis [6], the most characterized cell death pathway in the last decade [7]. In particular, necroptosis is triggered by the activation of RIPK1, TRIF, or ZBP1, and requires the RIPK3-dependent phosphorylation of MLKL. Then, the oligomerization of MLKL results in plasma membrane rupture, leading to a lytic form of cell death [8]. The essential role of necroptosis is confirmed in many clinical manifestations, including inflammation, cancer, neurological diseases, and ischemia-reperfusion injury [9,10,11].

As a specific inhibitor of RIPK1, necrostatin-1 (Nec-1) showed a protective effect on ischemia-reperfusion injury via inhibiting RIPK1 autophosphorylation, thereby blocking the necroptosis pathway [12]. For example, Nec-1 improves cardiac function by inhibiting myocardial tissue necroptosis in the acute myocardial infarction model [13]; similarly, Nec-1 reduced necroptosis of tubular cells and resulted in significant survival benefits in a lethal renal IR injury model [14]. While in addition to necroptosis, RIPK1 involves apoptosis and inflammation, and its specific role depends on the cell type and disease setting [12,15]. Thus, RIPK1 inhibitor Nec-1 showed anti-apoptosis and anti-inflammatory effects in specific conditions in addition to inhibiting necroptosis [16]. Previous studies demonstrated that Nec-1 improved lung function and reduced the inflammation response in LIRI [17,18]; however, the underlying mechanism of Nec-1 in LIRI remains unclear. LIRI is a complex pathophysiological process involving multiple signaling pathways and cell types [19]; therefore, understanding the effects of Nec-1 will provide new mechanistic insight into LIRI.

In this study, we established an in vitro cold ischemia-reperfusion model in BEAS-2B cells, which mimics the lung transplantation setting, and an in vivo mouse lung hilar clamping-induced lung ischemia-reperfusion model. We assessed the effects of Nec-1 on these models and further explored its action mechanism.

## 2. Materials and Methods

### 2.1. Animals, Cell Lines, and Reagents

C57BL/6 J mice (male, 8–10 weeks, 25–28 g) were purchased from the Experimental Animal Center (SCXK (Zhe) 2018-0006), Zhejiang University (Hangzhou, China). The mice are free to access water and food in an air-conditioned room (22–24 °C, relative humidity 50%) on a 12 h light/dark cycle.

BEAS-2B cells (CRL-9609) purchased from ATCC (American Type Culture Collection, Manassas, VA, USA) were cultured in low-glucose Dulbecco’s modified Eagle’s medium (DMEM, Gibco C11885500BT, Thermo Fisher Scientific, Waltham, MA, USA) supplemented with 10% fetal bovine serum (FBS, Gibco 10099141, Thermo Fisher Scientific, Waltham, MA, USA) with 5% CO_2_ at 37 °C. The medium was renewed every two to three days until cell confluence. Then, the cells were detached with 0.25% trypsin–0.02% EDTA (T6539, Macklin Inc., Shanghai, China) and were seeded at 15,000 cells/well on 96-well plates, at 4 × 10^5^ cells/well on 6-well plates and at 6 × 10^4^ cells/well on 24-well plates.

Necrostatin-1 (Nec-1, 586587, J&K Scientific Ltd., Beijing, China) was dissolved in dimethyl sulfoxide (DMSO, D8418, Sigma-Aldrich, Merck, Kenilworth, NJ, USA) and diluted in a culture medium or saline before use (the concentration of DMSO was lower than 0.3% finally).

### 2.2. Animal Model and Pharmacological Treatment

Lung transient ischemia was induced by applying a microvascular clamp on the left hilum of the mouse for 1 h, and then the clamp was released for reperfusion, as reported previously [20]. Four treatments were performed in these mice: sham-operated vehicle control (Sham, *n* = 12), sham-operated Necrostatin-1 control (Nec-1, 1 mg/kg, *n* = 12), left lung ischemia-reperfusion injury operation with vehicle (LIRI, *n* = 12), and left lung ischemia-reperfusion injury operation with Nec-1 (LIRI + Nec-1 1 mg/kg, *n* = 12). Mice were pretreated with vehicle or vehicle-containing Nec-1 via intraperitoneal injection (i.p., 1 mg/kg) 1 h before the ischemia operation. These mice were sacrificed at 2 h of reperfusion, and lung tissues were sampled for pathological and biochemical experiments. All analysis was performed by an examiner who did not know the study design.

### 2.3. Simulated IR Model for Lung Transplantation and Pharmacological Treatment

A cellular model was made to simulate the cold-ischemia/reperfusion (CI/R) procedure during lung transplantation in clinical [21]. Briefly, BEAS-2B cells were rinsed twice with cold PBS and then incubated with 4 °C Perfadex^®^ lung preservation solution. Then, the cultured cells were placed in a 4 °C chamber filled with 50% O_2_ for the indicated time (6 h, 12 h, 18 h, or 24 h). At the same time, controls were similarly washed and cultured in normal conditions. At the end of mimicked ischemia, the cultures were returned to the standard culture condition for the indicated time (0 h, 2 h, or 4 h) for reperfusion. Nec-1 was added at the onset of CI/R and was sustained during the whole CI/R procedure in BEAS-2B cells.

### 2.4. Hematoxylin and Eosin (HE) Staining and Pathological Evaluation

The left lungs of mice were fixed for 48 h in 4% paraformaldehyde and then embedded in paraffin as described previously [22]. Lung sections were cut at 4 μm from paraffin-embedded lung tissues, and HE staining was performed to indicate inflammatory changes in mouse lungs after ischemia-reperfusion treatment. Tissue morphological changes were observed under a microscope and photographed (Olympus BX51, Olympus Corporation, Tokyo, Japan). A blinded examiner evaluated the lung injury using a scoring system described previously [23]. According to the previous reports [18], the epithelial thickness of small airways with similar diameters, perivascular cuff area and vessel area were measured using software (ImageJ, Version 1.37c, Bethesda, MD, USA). Additionally, the lung epithelial thickness and vessel edema evaluation were analyzed by an analyst blinded to the treatments.

### 2.5. Immunohistochemical (IHC) and Immunofluorescence (IF) Staining

Lung sections (4 μm thickness) were immersed in 3% H_2_O_2_ for 25 min and then blocked in 3% BSA for 30 min at room temperature. After that, the sections were incubated with anti-phospho MLKL(S345) antibody (#62233, Cell Signaling Technology, Danvers, MA, USA) overnight at 4 °C. These sections were incubated with secondary antibody conjugated with HRP (1:1, K5007, Dako, Glostrup, Denmark) for 50 min at next day. Then, the sections were washed 3 times with PBS (pH = 7.4) and exposed to DAB (0.05% 3,3-diaminobenzidine, K5007, Dako, Denmark) for 2 min. The sections were observed and photographed under a microscope (Olympus BX51, Olympus Corporation, Tokyo, Japan). The expression of phospho-MLKL (pMLKL) in biopsy sections was analyzed immunohistochemically to quantify the ratio of the pMLKL-positive area to the whole lung tissue area (pMLKL area).

Immunofluorescence staining was performed on paraffin-embedded tissue slices. Co-staining of phospho-MLKL (#62233, Cell Signaling Technology, Danvers, MA, USA) and SPC (ab211326, Abcam, Cambridge, UK) was performed using a three-color Fluorescence kit (Recordbio Biological Technology, Shanghai, China) based on the tyramide signal amplification (TSA) technology according to the manufacturer’s instruction [23]. The slices were observed and photographed under a microscope (Olympus BX51, Olympus Corporation, Tokyo, Japan). The quantification of immunofluorescence and immunohistochemistry was performed with ImageJ software (Version 1.37c, Bethesda, MD, USA).

### 2.6. Detection of Cytokines in Bronchoalveolar Lavage Fluid (BALF) of Mice and BEAS-2B Cell Culture Supernatants

After IR treatments, 0.5 mL PBS was used to obtain BALF from the left lungs of the mice. Then, BALF was centrifuged (1000× *g*, 4 min) to remove cell debris. Protein concentrations of the BALF were determined using a BCA protein assay kit (P0011, Beyotime Biotechnology, Shanghai, China).

After CI/R treatments, medium samples (1000 μL) were collected and stored at −40 °C. The cells were lysed at 4 °C in lysis buffer (Kangchen Biotechnology, Shanghai, China) for 30 min and then centrifugated at 1200× *g* for 10 min. Protein concentrations were determined by BCA assay.

The concentration of TNF-α and IL-6 in the BALF and in the cell culture medium was detected using commercial ELISA kits (EK0527, mouse TNF-α ELISA kit; EK0411, mouse IL-6 ELISA kit; EK0410, human TNF-α ELISA kit; EK0525, IL-6 ELISA kit; Wuhan Boster Biological Technology, Ltd., Wuhan, China). The detections were performed according to the manufacturer’s instructions. The results were calculated and reported as pg/mg protein or pg/mL.

### 2.7. Quantitative Real-Time Polymerase Chain Reaction (RT-PCR) Analysis

At the end of treatment, total RNA was isolated from mouse lung tissues or BEAS-2B cells with RNAiso PLUS reagent (Takara Biotechnology Co., Ltd., Dalian, China) according to the manufacturer’s instructions. The primers used are listed in Table S1. For BEAS-2B cells and mouse lung tissues, GAPDH and β-actin were the internal control, respectively. The RNA amplification kit SYBR^®^ Premix Ex Tag™ II (Takara Biotechnology Co., Ltd., Dalian, China) was used. LightCycler^®^ 480 II system (Roche, Alameda, CA, USA) was used for qRT-PCR performance. The relative quantification of mRNA expression was calculated according to the 2^−ΔΔCt^ method, where Ct is the cycle threshold,

ΔCt = Ct_target_ − Ct_β-actin_,

and

ΔΔCt = ΔCt_treatment_ − ΔCt_control_.

### 2.8. Western Blotting Analysis

Lung tissue was sampled and shredded, then 4 °C NP-40 lysis buffer (P0013F, Beyotime Biotechnology, Shanghai, China) containing 1 mM PMSF (ST506, Beyotime Biotechnology, Shanghai, China), protease and phosphatase inhibitor cocktail (P1049, Beyotime Biotechnology, Shanghai, China) was added with 100 μL lysis buffer for 10 mg tissue. Then, the soluble part of the protein lysate was obtained by centrifugation at 12,000× *g* at 4 °C for 30 min. The precipitation was further lysed with the above NP-40 lysis buffer containing 6 M urea (ST1731, Beyotime Biotechnology, Shanghai, China) to obtain the insoluble part of the protein lysate.

BEAS-2B cells were washed twice with ice-cold PBS and then lysed at 4 °C in NP-40 lysis buffer containing 1 mM PMSF, protease and phosphatase inhibitor cocktail. The lysate was obtained by centrifugation at 12,000× *g* at 4 °C for 30 min.

Protein concentrations were determined using a BCA protein assay kit (P0011, Beyotime Biotechnology, Shanghai, China). Protein samples (50–80 μg) were applied for Western blotting. The following antibodies were used: mouse monoclonal antibody against RIPK1 (1:1000, ab56164, Abcam, Cambridge, UK), rabbit polyclonal antibody against RIPK3 (1:1000, ab56164, Abcam, Cambridge, UK), rabbit polyclonal antibody against phosphor-RIPK3 (S227, 1:1000, ab209384, Abcam, Cambridge, UK), rabbit polyclonal antibody against MLKL (1:1000, ab79823, Abcam, Cambridge, UK), rabbit polyclonal antibody against phosphor-MLKL (S358, 1:1000, ab187091, Abcam, Cambridge, UK), rabbit polyclonal phosphor-RIPK1 antibody (S166, 1:1000, #44590, Cell Signaling Technology, Danvers, MA, USA), mouse monoclonal antibody against Phospho-IκB (1:1000, #9246, Cell Signaling Technology, Danvers, MA, USA), rabbit Polyclonal Antibody against cleaved caspase 3 antibody (1:1000, #9661, Cell Signaling Technology, Danvers, MA, USA), rabbit polyclonal antibody against IκBα (1:1000, AF5204, Beyotime Biotechnology, Shanghai, China), rabbit monoclonal antibody against Bax (#CPA1092, Cohesion Bio, London, UK), rabbit monoclonal antibody against cleaved caspase 8 (#WL00659, Wanleibio, Shenyang, China), and mouse monoclonal antibody against glyceraldehyde-3-phosphate dehydrogenase (GAPDH, 1:5000, #60004-1-Ig, Proteintech, Wuhan, China). The secondary antibody was HRP-conjugated goat anti-mouse IgG (1:5000, #7076S, Cell Signaling Technology, Danvers, MA, USA) or HRP-conjugated goat anti-rabbit IgG (1:10000, 111-035-003, Jackson, West Grove, PA, USA). The immunoblots were detected with a G-Box Multi fluorescence and chemiluminescence imaging system (Syngene, Frederick, MD, USA). The blot intensity of the detected protein was analyzed using software (imageJ, Version 1.37c, Bethesda, MD, USA) and normalized by internal control (GAPDH) for each sample.

### 2.9. Assessment of Cell Viability, Cell Injury, and Cell Death

The cell viability was determined using Trypan blue exclusion test. Trypan blue staining is used to label dead cells and quantify live cells exclusively. At the end of CI/R, the cells were detached with trypsin/EDTA and resuspended in the culture medium mixed with 4% trypan blue (1:1, T8154, Sigma-Aldrich, Merck, Kenilworth, NJ, USA). The trypan blue negative cells were counted under a microscope using a hemocytometer. The results are reported as the percentage of negative staining live cells in total cells.

Cell injury was quantified using lactate dehydrogenase (LDH) activity assay in the cell medium. The LDH activity assay was carried out by a colorimetric method using a commercial kit (A020-1, JianCheng Biotechnology Co., Ltd., Nanjing, China). The result was calculated as follows:

net percentage of LDH release = 100% × (stimulated release − spontaneous release)/(total release − spontaneous release)

Results are reported as percentages of control.

For detection of cell death, BEAS-2B cells grown on the coverslips were stained with 10 μg/mL of propidium iodide (PI, ST511, Beyotime Biotechnology, Shanghai, China) and 10 μg/mL of Hoechst 33258 (C1011, Beyotime Biotechnology, Shanghai, China) at the end of CI/R for 10 min at 37 °C. Then, the cells were checked and photographed under a fluorescent microscope (Olympus BX51, Olympus Corporation, Tokyo, Japan). The apoptotic cells were determined as fragmented or condensed nuclei with bright Hoechst staining, and the necrotic cells were determined as condensed nuclei with red PI staining. At least 1000 cells were counted in five different fields in one coverslip. The results were reported as percentages of apoptotic or necrotic cells in total cells.

### 2.10. Immunofluorescent Analysis of HMGB1 Distribution, and Colocalization of RIPK1 and RIPK3

After the treatment of cold ischemia for 18 h and reperfusion for the indicated time, BEAS-2B cells on coverslips were washed with PBS and then fixed with 4% paraformaldehyde for 15 min. The cells were washed with PBS containing 0.1% Triton X-100 three times (10 min once) and incubated with 5% normal donkey serum for 30 min at room temperature. The immunofluorescent staining of HMGB1, RIPK1, and RIPK3 in cells was performed by incubation with rabbit polyclonal antibody against HMGB1 (1:1000, ab79823, Abcam, Cambridge, UK), mouse monoclonal antibody against RIPK1 (1:600, ab72139, Abcam, Cambridge, UK), and rabbit polyclonal antibody against RIPK3 (1:600, ab56164, Abcam, Cambridge, UK) overnight at 4 °C. The slides were incubated with Cy3-conjugated donkey anti-rabbit IgG (1:200, AP182C, Sigma-Aldrich, Merck, Kenilworth, NJ, USA) or FITC-conjugated goat anti-mouse IgG (1:200, AP181F, Sigma-Aldrich, Merck, Kenilworth, NJ, USA) at room temperature for 2 h. Finally, the coverslips were mounted using ProLong^TM^ Gold Antifade Mountant (Invitrogen, P10144, Thermo Fisher Scientific, Waltham, MA, USA), and images were captured under an Olympus fluorescent microscope (Olympus BX51, Olympus Corporation, Tokyo, Japan) and analyzed using software (ImageJ, Version 1.37c, Bethesda, MD, USA).

### 2.11. Flow Cytometry

After harvested, left lung tissues were mechanically dissociated and subsequently digested in DMEM supplemented with DNase I and collagenase XI at 37 °C for 40 min. Cells were passed through 70 μm cell strainers, and erythrocytes were lysed using red blood cell lysis buffer (C3702, Beyotime Biotechnology, Shanghai, China). To reduce non-specific binding, suspensions were pre-incubated with an Fc receptor blocking solution (#156603, Biolegend, San Diego, CA, USA). Then, cells were stained with fluorochrome-conjugated antibodies for 30 min on ice, washed, and resuspended with PBS. The neutrophil was identified by anti-Ly6G-Pacific Blue (#127612, Biolegend, San Diego, CA, USA) and anti-CD11b-FITC (#101205, Biolegend, San Diego, CA, USA); macrophage was identified by anti-F4/80-BV650 (#563402, BD Bioscience, CA, USA) and anti-CD45-Alexa Fluor 700 (#560510, BD Bioscience, CA, USA). According to the manufacturer’s protocol, the apoptosis of neutrophils and macrophages was determined using an Annexin V-APC apoptosis detection kit (Beijing 4 A Biotech, Beijing, China). Finally, samples were acquired in a CytoFLEX LX flow cytometer (Beckman Coulter, Brea, CA, USA) and were analyzed using FlowJo software (TreeStar, Ashland, OR, USA).

### 2.12. Statistical Analysis

Data are presented as mean ± SD and analyzed using GraphPad Prism Software (version 4.0, GraphPad Software Inc., San Diego, CA, USA). ‘*n*’ indicates the number of mice used for the experiments or independent cell culture preparations. Grubbs’ test was used to identify outliers. D’Agostino&Pearson omnibus normality test was performed to test the normality of the data. To assess the equal variances of the data Brown–Forsythe test was performed. When the data passed the normality and equal variance test, one-way ANOVA was performed to assess the difference among means. A student’s *t*-test was performed to assess the difference between the two means. When the data did not pass the normality or equal variance test, we used nonparametric statistics (Kruskal–Wallis test to assess the difference among medians and Mann–Whitney test to assess the difference between the two medians). A value of *p* < 0.05 was considered statistically significant [24].

## 3. Results

### 3.1. Necrostatin-1 Alleviated Lung Injury and Inflammation Caused by Ischemia Reperfusion in Mice

**Figure 1:**
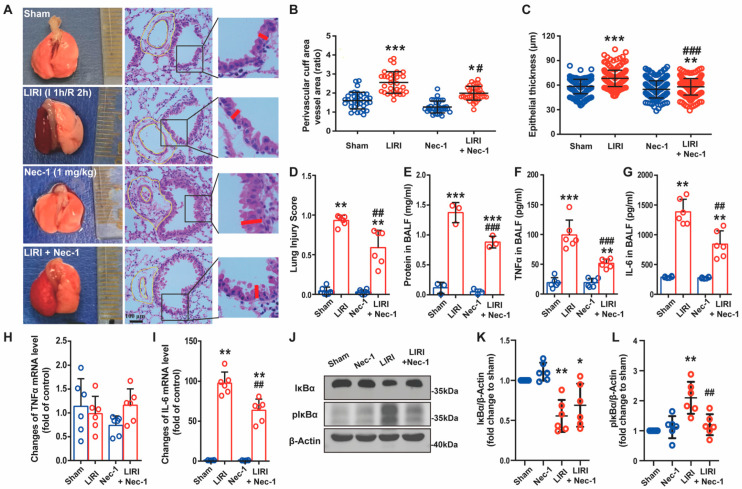
Necrostatin-1 alleviated lung injury and inflammatory response caused by ischemia reperfusion (IR) in mice. The lung ischemia-reperfusion injury (LIRI) was induced by clamping the left pulmonary hilum of the mouse for 1 h using a microvascular clip and then removing the clip for reperfusion for 2 h. Nec-1 (1 mg/kg) was administered intraperitoneal injection 1 h before ischemia. (**A**) Changes in the gross appearance of mouse lungs and the representative HE staining of the lungs. The area between the two yellow cycle was determined as the perivascular cuff area, and the red line indicated the thickness of the epithelium. (**B**) The ratio of perivascular cuff area to vessel area. (**C**) The thickness of the epithelial barrier. (**D**) Lung injury score. (**E**) Total protein exudation in the bronchoalveolar lavage fluid (BALF). (**F**,**G**) The concentration of TNFα and IL-6 in the BALF detected using ELISA. (**H**,**I**) The mRNA level of TNFα and IL-6 determined by real-time PCR. (**J**) The level of IκB and phosphor-IκB in lung tissue homogenates detected by Western blotting. (**K**,**L**) Quantification of proteins from Western blots in panel J. *n* = 6 animals per group. ^*^ *p* < 0.05, ^**^ *p* < 0.01, ^***^ *p* < 0.001, compared with Sham; ^#^ *p* < 0.05, ^##^ *p* < 0.01, ^###^ *p* < 0.001, compared with LIRI; one-way ANOVA.

LIRI was induced by clamping the left lung hilum for 1 h, followed by 2 h of reperfusion. Nec-1 was intraperitoneally administrated 1 h before the operation at a dose of 1 mg/kg. From the gross view and HE staining, the mouse lungs experienced severe injury after IR and partially reversed by Nec-1 administration (Figure 1A). Compared with the sham, the mouse lung exhibited severe perivascular edema (Figure 1B, the ratio of perivascular cuff area to vessel area) and terminal airway epithelium swelling (Figure 1C, the thickness of epithelium) after IR, both of which alleviated by Nec-1. The reduction in the lung injury score also confirmed the efficacy of Nec-1 (Figure 1D). In addition, Nec-1 decreased the protein exudation in BALF, indicating Nec-1 inhibited the destruction of the pulmonary blood–air barrier caused by IR (Figure 1E). To explore whether Nec-1 reduced the inflammatory responses, the change in TNFα and IL-6 in transcriptional and translational levels were tested. Both TNFα and IL-6 levels in BALF increased after IR and then decreased with Nec-1 administration (Figure 1F,G). Moreover, Nec-1 reduced the increase in IL-6 mRNA level, but it showed almost no changes in TNFα mRNA level with or without IR (Figure 1H,I). Meanwhile, IR increased the expression of phosphor-IκB, and Nec-1 abrogated the increment, which reflected that the activated NF-κB pathway was inhibited by Nec-1 (Figure 1J,L). Thus, these results suggested that Nec-1 alleviated LIRI and inflammatory response in mice.

### 3.2. Necrostatin-1 Inhibited IR-Induced Necroptosis of Lung Epithelial Cells in Mice

**Figure 2:**
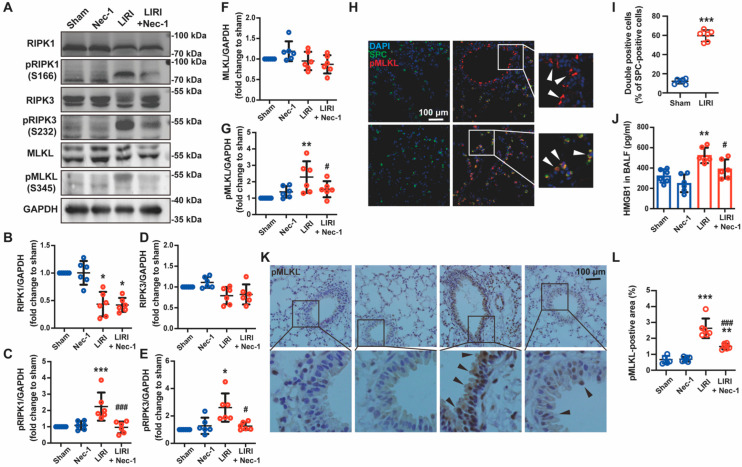
Necrostatin-1 inhibited IR-induced necroptosis of lung epithelial cells in mice. The mouse LIRI was induced by clamping the left pulmonary hilum of the mouse for 1 h using a microvascular clip and then removing the clip for reperfusion for 2 h. Nec-1 (1 mg/kg) was administered intraperitoneal injection 1 h before ischemia. (**A**) Western blotting of necroptosis-related proteins (RIPK1, phosphor-RIPK1, RIPK3, phosphor-RIPK3, MLKL, and phosphor-MLKL) in lung tissue homogenates. (**B**–**G**) Quantification of proteins from Western blots in panel A. (**H**) Immunofluorescence staining of phosphor-MLKL and SPC (a marker of lung epithelial cell) in peripheral bronchial epithelium (upper panel) and alveolar epithelium (lower panel). White arrows indicated that pMLKL was localized at airway or alveolar epithelial cells. (**I**) Percentage of SPC and pMLKL double-positive cells in SPC positive cells. (**J**) The concentration of HMGB1 in BALF detected using ELISA. (**K**) Immunohistochemical staining of phosphor-MLKL in different groups. Black arrows indicated the pMLKL-positive areas in the bronchial epithelium. (**L**) The ratio of the pMLKL-positive area to the whole lung tissue area. *n* = 6 animals per group. ^*^ *p* < 0.05, ^**^ *p* < 0.01, ^***^ *p* < 0.001, compared with Sham; ^#^ *p* < 0.05, ^##^ *p* < 0.01, ^###^ *p* < 0.001, compared with LIRI; one-way ANOVA.

We then explored whether Nec-1 alleviates LIRI by blocking necroptosis. Immunoblotting showed that ischemia reperfusion activated the necroptosis pathway in the mouse lung (Figure 2A). Specifically, necroptosis initiator phosphor-RIPK1, key molecule phosphor-RIPK3, and executor phosphor-MLKL dramatically increased when the mouse lung underwent IR, and Nec-1 almost reversed the phosphorylation of these molecules (Figure 2B–G). The cell type that experienced necroptosis in lung tissue was then identified using phosphor-MLKL immunofluorescence staining. The expression of phosphor-MLKL markedly increased after IR and was predominantly located in airway epithelial cells and alveolar epithelial cells (Figure 2H,I). Meanwhile, HMGB1, an endogenous damage-associated molecular pattern (DAMP) released by necrotic cells, was detected using ELISA. We found that Nec-1 reduced the release of HMGB1 caused by IR in BALF (Figure 2J). As shown in Figure 2H, the immunohistochemical analysis of phosphor-MLKL also confirmed that the necroptotic cells were epithelial cells and further indicated that Nec-1 alleviated necroptosis induced by IR (Figure 2K,L). These findings demonstrated that Nec-1 alleviated LIRI by inhibiting the necroptosis of epithelial cells.

### 3.3. Necrostatin-1 Mitigated Cold Ischemia and Reperfusion-Induced Cell Death and Production of Inflammatory Cytokines in BEAS-2B Cells

**Figure 3:**
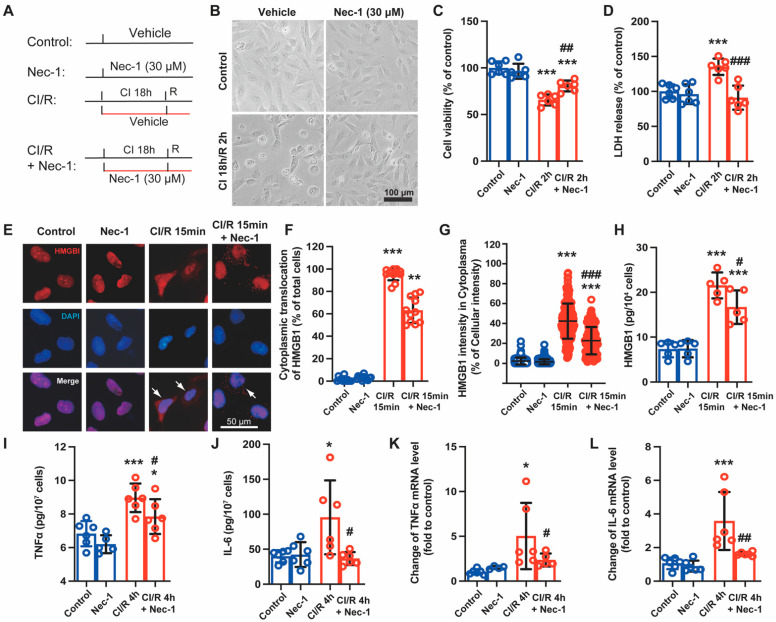
Necrostatin-1 mitigated cold ischemia and reperfusion (CI/R)-induced cell death and production of inflammatory cytokines in BEAS-2B cells. BEAS-2B cells were incubated in a 4 °C chamber filled with 50% oxygen for 18 h with ice-cold lung preservation Perfadex^®^ solution to simulate the procedure of clinical donor lung preservation and then subjected to a warm culture medium to mimic reperfusion. Necrostatin-1 (Nec-1, 30 μM) was added to the medium at the beginning of cold-ischemia and sustained during reperfusion. (**A**) Cell experiment protocol. (**B**) Morphological changes of BEAS-2B cells in different groups. (**C**) Cell viability determined using trypan blue exclusive staining. (**D**) LDH in the cell culture medium. (**E**) The distribution of HMGB1 in the cells observed by immunofluorescent staining. (**F**) The counts of cells undergoing HMGB1 cytoplasmic translocation. The percentage of the cells with HMGB1 cytoplasmic translocation to total cells was calculated. (**G**) The percentage of the optical density of HMGB1 in the cytoplasm to that of the whole cell. (**H**–**J**) The level of HMGB1, TNFα and IL-6 in the cell culture supernatant determined using ELISA. (**K**,**L**) The mRNA level of TNFα and IL-6 detected using real-time PCR. Three independent experiments were performed. ^*^ *p* < 0.05, ^**^ *p* < 0.01, ^***^ *p* < 0.001, compared with Control; ^#^ *p* < 0.05, ^##^ *p* < 0.01, ^###^ *p* < 0.001, compared with CI/R. One-way ANOVA.

As showed that IR caused significant necroptosis in lung epithelial cells, we then used BEAS-2B cells, immortalized human bronchial epithelial cells, to explore the effect of Nec-1 in a cold ischemia-reperfusion (CI/R) cell model, which stimulated lung transplant setting [25]. The cells were subjected to cold ischemia for the indicated time (6, 12, 18, and 24 h) to mimic static storage with preservation solution and then subjected to a warm culture medium to mimic reperfusion for different time intervals (0, 2, and 4 h) (Figure S1). The cells exhibited rounding and swelling in the prolongation of cold ischemia time and shrinking after reperfusion (Figure S1A). With the extension of cold ischemia and reperfusion time, trypan blue-positive cells increased significantly, while the number of viable cells decreased (Figure S1B–E). Based on the change of cell viability with time, we used ischemia for 18 h and reperfusion for 2 h (CI 18 h/R 2 h) as the main injury condition. The preliminary experiment showed that Nec-1 at 30 μM significantly reversed CI/R-induced cell viability reduction in BEAS-2B cells (Figure S2A,B). According to the results of different administration regimes (Figure S2C,D), Nec-1 was added at the beginning of cold ischemia and sustained during reperfusion in the following experiments (Figure 3A). Nec-1 improved CI/R-induced cell morphological changes (Figure 3B), reversed the cell viable reduction (Figure 3C), and decreased cell damage caused by CI/R (Figure 3D). Then, we tested the HMGB1 translocation from nuclear to cytoplasmic and its release in BALF, both of them indicating cell necrosis. Immunofluorescence staining showed that after cold ischemia for 18 h and reperfusion for 15 min, HMGB1 significantly translocated from nuclear to cytoplasm in BEAS-2B cells, suggesting cell necrosis occurred early after reperfusion, and this translocation of HMGB1 was reduced by Nec-1 (Figure 3E–G). Furthermore, the ELISA assay showed that Nec-1 decreased the release of HMGB1 from BEAS-2B cells treated with CI/R 2 h (Figure 3H). At CI 18 h/R 4 h, TNFα and IL-6 increased in the cellular supernatant (Figure 3I,J); similarly, the mRNA synthesis of TNFα and IL-6 was promoted. Both the synthesis and release of these inflammatory cytokines were reversed by Nec-1 to some extent (Figure 3K,L).

### 3.4. Necrostatin-1 Reduced CI/R-Induced Cell Death through Inhibition of RIPK1-Dependent Apoptosis and Necroptosis in BEAS-2B Cells

**Figure 4:**
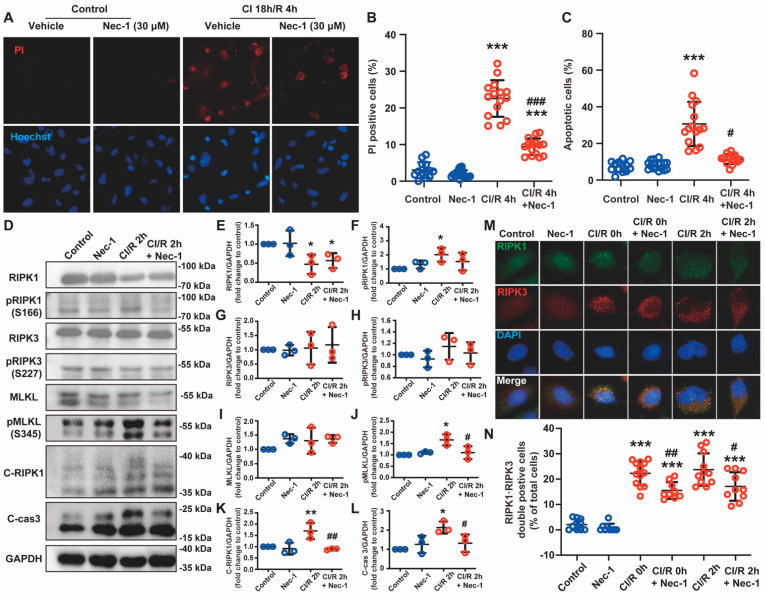
Necrostatin-1 reduced CI/R-induced cell death through the inhibition of RIPK1-dependent apoptosis and necroptosis in BEAS-2B cells. BEAS-2B cells were incubated in a 4 °C chamber filled with 50% oxygen for 18 h with ice-cold lung preservation Perfadex^®^ solution to simulate the procedure of clinical donor lung preservation and then subjected to a warm culture medium to mimic reperfusion. Necrostatin-1 (Nec-1, 30 μM) was added to the medium at the beginning of cold-ischemia and sustained during reperfusion. (**A**) Propidium iodide (PI) and Hoechst staining in BEAS-2B cells after CI/R. (**B**,**C**) Quantification of necrotic and apoptotic cells in panel A. (**D**) Western blotting of necroptosis- and apoptosis-related proteins (RIPK1, phosphor-RIPK1, RIPK3, phosphor-RIPK3, MLKL, phosphor-MLKL, cleaved RIPK-1, and Cleaved caspase 3) in BEAS-2B cells. (**E**–**L**) Quantification of proteins from Western blots in panel D. (**M**) The colocalization of RIPK1 and RIPK3 determined by immunofluorescence staining. (**N**) Cell counting of the RIPK1 and RIPK3 double-positive cells. Three independent experiments were performed. ^*^ *p* < 0.05, ^**^ *p* < 0.01, ^***^ *p* < 0.001, compared with Control; ^#^ *p* < 0.05, ^##^ *p* < 0.01, ^###^ *p* < 0.001, compared with CI/R. One-way ANOVA.

Then, we explored how Nec-1 reduced CI/R-induced cell death in BEAS-2B cells. We confirmed the effect of Nec-1 on CI/R-induced apoptosis and necrosis by a double immunofluorescence staining. As determined by PI staining and Hoechst staining (Figure 4A), CI 18 h/R 4 h induced significant necroptosis and apoptosis, and Nec-1 reversed both of them (Figure 4B,C). Immunoblotting showed that Nec-1 partially reversed the phosphorylation of RIPK1 and MLKL induced by CI/R, indicating the inhibitory effect of Nec-1 on necroptosis in BEAS-2B cells. Meanwhile, we observed that CI/R caused a significant decrease in RIPK1 and increased the protein level of cleaved RIPK1 (C-RIPK1) and cleaved caspase-3. All these changes were reversed by Nec-1, the specific inhibitor of RIPK1 (Figure 4D–L), which indicated that cells underwent RIPK1-dependent apoptosis [26,27]. The formation of insoluble necrosomes, RIPK1 and RIPK3 interacting through the RHIM domain, is essential to the occurrence of necroptosis [28]. Here, the immunofluorescence assay showed that both CI 18 h/R 0 h and CI 18 h/R 2 h induced RIPK1 and RIPK3 colocalized to form puncta in the cytoplasm, and Nec-1 significantly reduced the number of the cells with RIPK1 and RIPK3 double-positive puncta, which reflected that Nec-1 inhibited the formation of necrosome (Figure 4M,N). Thus, Nec-1 effectively inhibited the RIPK1-dependent apoptosis and necroptosis in the cold-ischemia and reperfusion model.

### 3.5. Necrostatin-1 Inhibited the Formation of Necrosome but Promoted Apoptosis in Mouse LIRI

**Figure 5:**
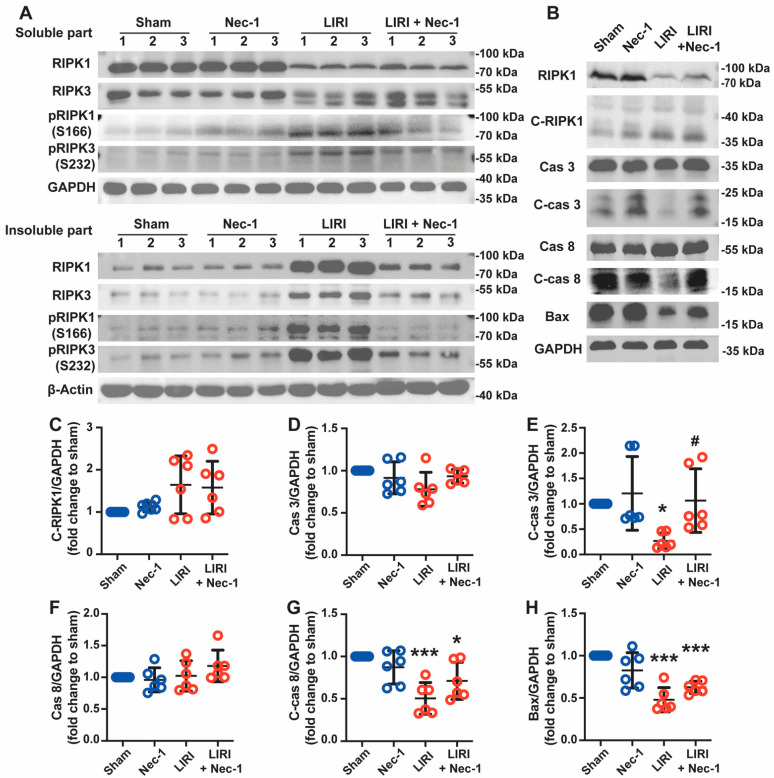
Necrostatin-1 inhibited the formation of necrosome but promoted apoptosis in mouse LIRI. The mouse LIRI was induced by clamping the left pulmonary hilum of the mouse for 1 h using a microvascular clip and then removing the clip for reperfusion for 2 h. Nec-1 (1 mg/kg) was administered intraperitoneal injection 1 h before ischemia. (**A**) Western blotting of RIPK1, phosphor-RIPK1, RIPK3, and phosphor-RIPK3 in soluble parts (upper panel) and insoluble parts (lower panel) of lung tissue homogenates. *n* = 3 animals per group. (**B**) Western blotting of apoptosis-related proteins (cleaved RIPK1, caspase 3, cleaved caspase 3, caspase 8, cleaved caspase 8, and Bax) in lung tissue homogenates. (**C**–**H**) Quantification of proteins from Western blots in panel B. *n* = 6 animals per group. ^*^ *p* < 0.05, ^**^ *p* < 0.01, ^***^ *p* < 0.001, compared with Sham; ^#^ *p* < 0.05, ^##^ *p* < 0.01, ^###^ *p* < 0.001, compared with LIRI; One-way ANOVA.

We further explored whether Nec-1 alleviated LIRI through the inhibition of the formation of necrosomes and RIPK1-mediated apoptosis in vivo. As shown in Figure 5A, RIPK1 and RIPK3 moved from soluble fraction into insoluble fraction after IR, and Nec-1 vastly decreased these movements, indicating the inhibitory effect of Nec-1 on the formation of necrosomes. Nec-1 also inhibited the phosphorylation of RIPK1 and RIPK 3 caused by IR in both the soluble and insoluble parts. Thus, consistent with the results of in vitro, Nec-1 reversed the formation of necrosomes caused by IR in vivo.

However, Nec-1 did not affect cleaved-RIPK1 increasing induced by IR. Additionally, the expression of apoptosis-related proteins, including cleaved caspase-3, Bax, and cleaved caspase-8, significantly decreased after IR. These reductions were reversed by Nec-1, suggesting IR inhibited apoptosis, but Nec-1 promoted apoptosis during lung IR (Figure 5B–H). Thus, Nec-1 inhibited necrosome formation but promoted apoptosis during LIRI in mice.

### 3.6. Necrostatin-1 Promoted the Apoptosis of Neutrophils in Mouse LIRI

**Figure 6:**
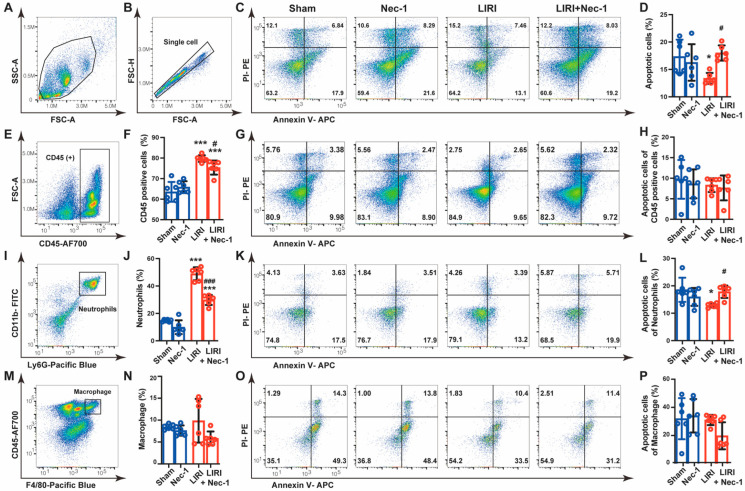
Necrostatin-1 promoted the apoptosis of neutrophils in mouse LIRI. The mouse LIRI was induced by clamping the left pulmonary hilum of the mouse for 1 h using a microvascular clip and then removing the clip for reperfusion for 2 h. Nec-1 (1 mg/kg) was administered intraperitoneal injection 1 h before ischemia. The left lung tissues were mechanically dissociated and subsequently digested, and then the single-cell suspensions were prepared from lung tissue. (**A**,**B**) Gating strategy delineating mouse lung single-cell suspensions. (**C**) Annexin V and PI staining of single cells prepared from mice lungs. Apoptotic cells were identified by Annexin V positive but PI negative cells. (**D**) Apoptosis rates of single-cell suspension in lung tissues. (**E**) Gating strategy delineating lung CD45+ subsets. (**F**) Percentage of CD45+ cells in total cells isolated from lung tissues. (**G**) Annexin V and PI staining of CD45+ cells in mice lungs. (**H**) Apoptosis rates of CD45+ cells. (**I**) Gating strategy delineating lung neutrophils subsets (CD11b + Ly6G + cells) in lung tissues. (**J**) Percentage of neutrophils in total cells isolated from lung tissues. (**K**) Annexin V and PI staining of neutrophils in mice lungs. (**L**) Apoptosis rates of lung neutrophils. (**M**) Gating strategy delineating lung macrophage subsets (F4/80 + CD11b + cells). (**N**) Percentage of macrophages in total cells isolated from lung tissues. (**O**) Annexin V and PI staining of lung macrophages. (**P**) Apoptosis rates of lung macrophages. *n* = 6 animals per group. ^*^ *p* < 0.05, ^**^ *p* < 0.01, ^***^ *p* < 0.001, compared with Sham; ^#^ *p* < 0.05, ^##^ *p* < 0.01, ^###^ *p* < 0.001, compared with LIRI; one-way ANOVA.

Considering the inconsistent results that Nec-1 inhibited the apoptosis in epithelial cells (Figure 4) but promoted the apoptosis in lung tissue after IR (Figure 5), we speculated that Nec-1 might increase the apoptosis of other cell types in vivo. Innate immune cells, especially neutrophils, massive infiltrated and played significant roles in the LIRI [19]. Therefore, we tested the effects of Nec-1 on the main innate immune cells. Flow cytometry analysis demonstrated that Nec-1 reversed IR-caused apoptosis inhibition in single-cell suspensions prepared from lung tissue (Figure 6A–D), which was consistent with the expression change in apoptosis-related proteins from immunoblotting (Figure 5B). Then, we used CD45 as a marker to discriminate bone marrow-derived immune cells from lung parenchymal cells. The results showed that CD45-positive cells dramatically increased after IR, accounting for approximately 80% of total cells, and Nec-1 partially inhibited these increases. However, the apoptosis of CD45 positive cells was almost unchanged among different groups (Figure 6E–H). Ly6G and CD11b were then used as markers to indicate neutrophils in lung tissue. We observed that Nec-1 largely reversed the increase in neutrophils induced by IR. Notably, the apoptosis of neutrophils was decreased in the LIRI group, and Nec-1 promoted the apoptosis of neutrophils (Figure 6I–L). We further explored the effect of Nec-1 on macrophages. The results showed that macrophages, indicated by CD45 and F4/80, did not increase after IR, and Nec-1 had no effect on the apoptosis of macrophages (Figure 6M–P). These findings indicated that Nec-1 increased apoptosis of neutrophils in mouse LIRI.

## 4. Discussion

In the present study, we confirmed that Nec-1, the RIPK1 inhibitor, alleviated ischemia-reperfusion-induced cell death and inflammation both in vitro and in vivo. Nec-1 mitigated cell death via the inhibition of RIPK1-dependent apoptosis and the formation of necrosomes in BEAS-2B cells. In addition, Nec-1 reversed lung injury in mice by inhibiting the necroptosis of epithelial cells and promoting the apoptosis of neutrophils.

Our study showed that ischemia reperfusion caused a massive release of TNFα and activation of the necroptosis pathway, as well as the inhibitory effect of Nec-1, indicating ischemia reperfusion might result in TNFα-induced RIPK1-dependent necroptosis in lung tissues, rather than RIPK3-dependent necroptosis. Generally, the activation of RIPK3 is indispensable for necroptosis, and the kinase activity of RIPK3 can be induced by RIPK1, TRIF, or ZBP1 depending on different stimuli the cells received [6,8]. According to our results, IR-induced necroptosis in mouse lungs may initiate by binding TNFα to TNFR1, then recruiting and activating RIPK1. After that, activated RIPK1 combines with RIPK3 and thus leads to the formation of necrosome, followed by the phosphorylation of MLKL, finally leading to cell death [6,29]. Consistent with our results, a recent study found that Nec-1 obtained a comparable protective effect to RIPK3-deficient mice in the lung transplantation model [30], which further confirmed that the phosphorylation of RIPK1 contributed to LIRI. However, RIPK1 activation can mediate not only necroptosis but apoptosis and inflammation [31].

Indeed, we showed that cold ischemia reperfusion increased apoptosis and necroptosis, both of which were inhibited by Nec-1 in BEAS-2B cells, demonstrating that RIPK1-dependent necroptosis and apoptosis occurred under the same stimuli. The “co-existing” state of RIPK1-dependent necroptosis and apoptosis is theoretically possible, as it depends on the amount and the post-translational modifications (PTM) of RIPK1. The amount of RIPK1 in cells determines whether and how it recruits procaspase-8 and RIPK3, subsequently affecting cell fate [32]. Additionally, the complex post-translational modifications of RIPK1, including phosphorylation and ubiquitylation, govern whether RIPK1 involves the NF-κB, apoptotic, or necroptotic signaling pathway [33]. We believed that the amount of RIPK1 in epithelial cells induced by CI/R was sufficient to recruit subsequent molecules and activate RIPK1, while the mode and site of PTM of RIPK1 affected the proportion of cells undergoing necroptosis or apoptosis. Since necroptosis and apoptosis share the same upstream pathway and depend on the kinase activity of RIPK1, Nec-1 can inhibit both and mitigate damage. Similarly, the inhibitory effect of Nec-1 on both necroptosis and apoptosis has been reported in brain ischemia and osteoarthritis [34,35].

In addition to inhibiting cell death, we observed Nec-1 mitigated inflammation responses caused by IR in vitro and in vivo. This anti-inflammatory effect of Nec-1 is most likely achieved via its inhibition of cell death rather than RIPK1-mediated inflammation, as we showed that cell death occurred earlier than the inflammation during CI/R. The formation of necrosome and HMGB1 translocation from nuclear to cytoplasm were observed at the initiation of reperfusion, while the expression of inflammatory cytokines was much later than the changes in these markers of cell death. Thus, we believed that Nec-1 inhibited RIPK1-dependent cell death of epithelial cells, then decreased the released endogenous DAMPs, i.e., HMGB1, an activator of multiple inflammatory signal pathways, thereby mitigating inflammation responses. This process is also known as necroinflammation, which has been well documented in the pathogenesis of ischemia-reperfusion injury in various organs [36,37,38,39]. In addition, the off-target effect of Nec-1 should be considered. Nec-1 also inhibits indoleamine 2,3-dioxygenase (IDO), the enzyme of tryptophan catabolism, and is proven to be associated with neuroinflammation [40]. Whether Nec-1 alleviates lung inflammation by acting on IDO needs further studies.

Another interesting finding is that Nec-1 promotes the apoptosis of neutrophils, which is different from its effects on epithelial cells. The activation and infiltration of neutrophils play an essential role in LIRI [4,30]. The mechanism involved in the infiltration of mass neutrophils in LIRI is complex. It is widely accepted that damaged lung parenchymal release chemokines, which further activate and recruit neutrophils [19]. Additionally, the delayed apoptosis of neutrophils may also contribute to this. The timely apoptosis of neutrophils promotes the resolution of inflammation and avoids persistent injury to surrounding healthy tissues. The delayed apoptosis of neutrophils has been reported in ARDS patients [41], and Nec-1 specifically promoted neutrophil apoptosis in an ARDS animal model [42]. Here, we found Nec-1 induced the apoptosis of neutrophils in the LIRI model, and this pro-apoptotic effect may be related to the reduced release of TNFα by Nec-1. A previous study revealed that TNFα caused a delay in neutrophil apoptosis through the JNK/FoxO3a pathway and aggravated intestinal IR injury [43]. Therefore, we speculate that the effect of Nec-1 on promoting neutrophil apoptosis may be achieved through TNFα. Specifically, Nec-1 inhibited the death of lung parenchyma cells and the release of TNFα, which resulted in the amelioration of neutrophils’ delayed apoptosis under IR, and finally enhanced the resolution of inflammation and alleviated lung injury.

Our study has several limitations. First, although we scanned the concentration of Nec-1 in the cold ischemia-reperfusion model, the efficacy of Nec-1 in mouse LIRI was evaluated only with a pre-operation administration and a single dose. Second, the lung hilar ligation model is widely considered as the experimental model mimicking the mechanisms and pathological features of PGD [19,44]; however, it does not entirely represent human lung transplantation. Thus, further studies are needed to confirm the effects of Nec-1 on PGD through preclinical lung transplantation models.

## 5. Conclusions

In conclusion, we demonstrated that Nec-1 alleviated ischemia-reperfusion-induced acute lung injury and inflammation by inhibiting necroptosis and apoptosis of epithelial cells and promoting the apoptosis of neutrophils. Thus, Nec-1 appears to be a promising medication against the primary graft dysfunction of lung transplantation.

## Data Availability

The data presented in this study are available upon request from the corresponding author.

## Supplementary Materials

The following supporting information can be downloaded at: https://www.mdpi.com/article/10.3390/cells11193139/s1. Table S1: Primer pairs used for qRT-PCR; Figure S1: Simulated lung transplant setting in BEAS-2B cells; Figure S2: The administration regime of necrostatin-1 in BEAS-2B cells treated with ischemia reperfusion.
