# Supplementary material for: Necrostatin-1 Alleviates Lung Ischemia-Reperfusion Injury via Inhibiting Necroptosis and Apoptosis of Lung Epithelial Cells
Source: Cells. 2022 Oct 6;11(19):3139. doi: 10.3390/cells11193139 (PMC9563441; doi:10.3390/cells11193139)
Supplement: Supplementary file 1 [file cells-11-03139-s001.zip › cells-1871343-supplementary.pdf]

Supplemental Files

Table S1. Primer pairs used for qRT-PCR.

Name	Sequences (5' to 3')
GAPDH_*Homo*	F: GGAGCGAGATCCCTCCAAAAT
	R: GGCTGTTGTCATACTTCTCATGG
TNFα_*Homo*	F: CCTCTCTCTAATCAGCCCTCTG
	R: GAGGACCTGGGAGTAGATGAG
IL-6_*Homo*	F: ACTCACCTCTTCAGAACGAATTG
	R: CCATCTTTGGAAGGTTCAGGTTG
β-actin_*Mus*	F: GGCTGTATTCCCCTCCATCG
	R: CCAGTTGGTAACAATGCCATGT
TNFα_ *Mus*	F: CCTGTAGCCCACGTCGTAG
	R: GGGAGTAGACAAGGTACAACCC
IL-6_ *Mus*	F: CTGCAAGAGACTTCCATCCAG
	R: AGTGGTATAGACAGGTCTGTTGG

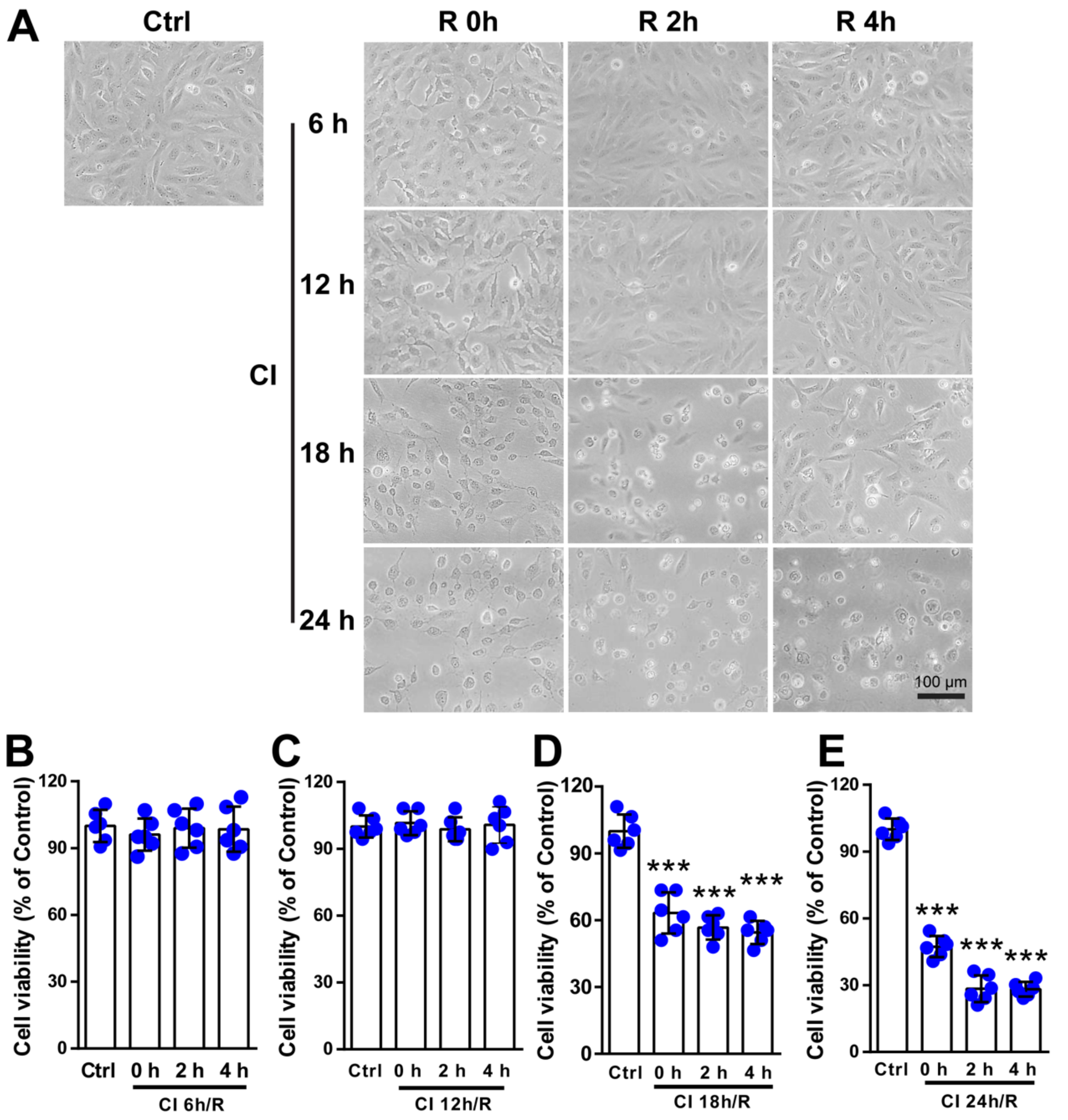

Figure S1. Simulated lung transplant setting in BEAS-2B cells.

(A) Typical morphological changes in BEAS-2B cells in different cold ischemia (CI) and reperfusion (R) time. Cells were treated with cold-ischemia (CI) for 6-24 hours and then with reperfusion(R) for 0-4 hours. **(B-E)** Cell viability determined using trypan blue exclusive staining in different groups. Six independent experiments were performed. ***P<0.001, compared with Control (Ctrl), One-way ANOVA.

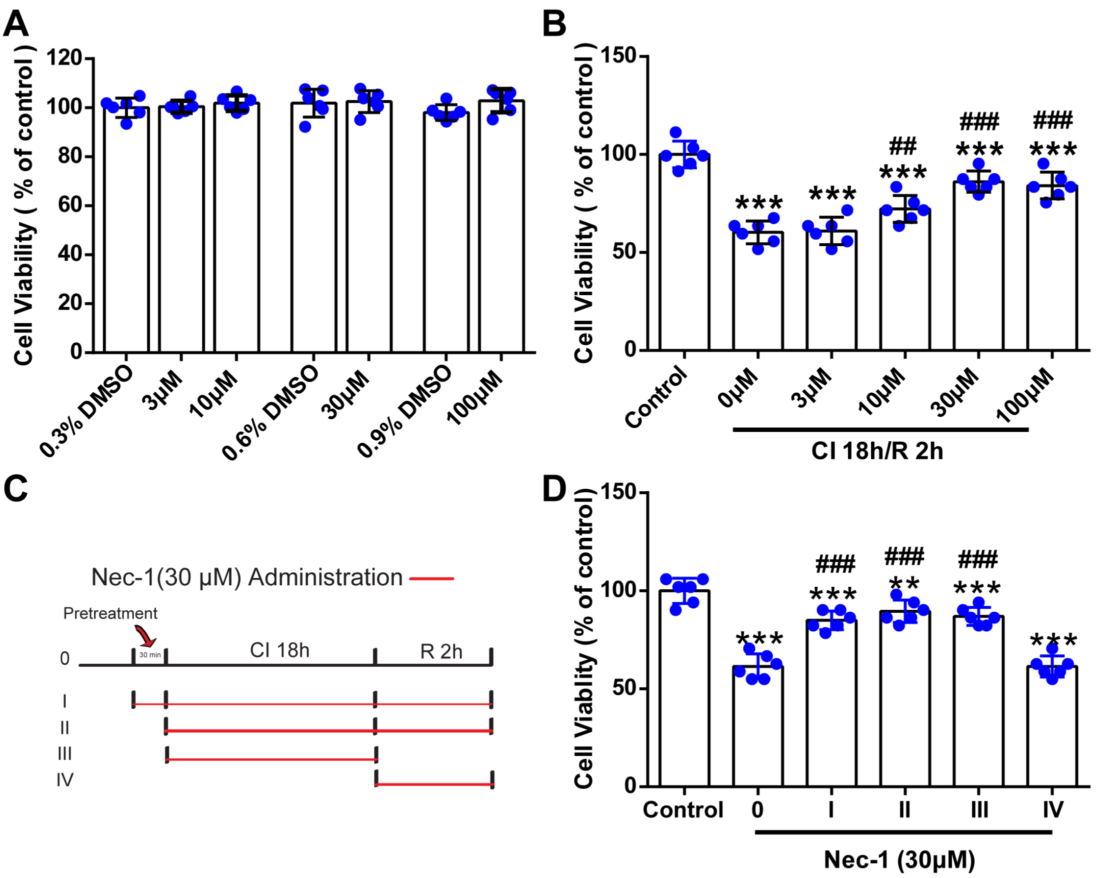

Figure S2. The administration regime of Necrostatin-1 in BEAS-2B cells treated with ischemia reperfusion. (A) Cell viability after Nec-1 or DMSO administration under normal condition. BEAS-2B cells were cultured in 96-well cell culture plate, the cell viability was determined using MTT assay. **(B)** Cell viability in different concentration of Nec-1 under CI/R condition. BEAS-2B cells treated with cold ischemia for 18 hours and reperfusion for 2 hours. Necrostatin-1 was administrated at the beginning of cold ischemia and sustained during reperfusion. **(C)** Different administration regime of Necrostatin-1. **(D)** Cell viability in different administration regime of Nec-1. BEAS-2B cells were cultured in 6-well cell culture plate. Necrostatin-1 (30 μM) was added according to the regime in which the BEAS-2B cells were treated with ischemia for 18 hours and reperfusion for 2 hours. Cell viability was determined using trypan blue exclusive staining. Six independent experiments were performed. **P<0.01, ***P<0.001, compared with control, $^{\#\#}$P<0.01, $^{\#\#\#}$P<0.001, compared with control, one-way ANOVA.
